# Prison Unhealthy Lifestyle and Poor Mental Health of Older Persons—A Qualitative Study

**DOI:** 10.3389/fpsyt.2021.690291

**Published:** 2021-11-19

**Authors:** Félix Pageau, Corinne Devaud Cornaz, Isabelle Gothuey, Helene Seaward, Tenzin Wangmo, Bernice S. Elger

**Affiliations:** ^1^Institute for Biomedical Ethics, University of Basel, Basel, Switzerland; ^2^Unit of Health Law and Humanitarian Medicine, Center for Legal Medicine, University of Geneva, Geneva, Switzerland

**Keywords:** lifestyle-related disease, mental health care, prison, physical activity, food

## Abstract

**Background:** Mental healthcare and lifestyle habits in prison, especially for older individuals, has been shown to be suboptimal. Most psychiatric conditions left untreated worsen food choices, physical inactivity, and substance abuse. In turn, bad habits lead to poorer mental health.

**Methods:** To comprehensively illustrate this downward spiraling, we completed a thorough analysis of data obtained through semi-structured qualitative interviews. There were 50 interviews of aging incarcerated people included in this article. They were analyzed following a classical six-stepped thematic analysis.

**Results:** According to our participants, sports are not well-adapted to aging individuals, nor to people with medical conditions. Prison is even more unadapted for those who both are aging and have medical conditions. Also, food served is less than optimal. According to our interviews, the older imprisoned individual often does not have access to food adapted to his or her medical conditions. Sport is maladapted for older incarcerated individuals and mostly tailored for younger ones. Finally, boredom and lack of responsibility hinder change toward a better lifestyle for older adults in prison.

**Conclusion:** Our paper shows why prison environmental modifications are needed to help older adults with their lifestyle habits. It also exposes an original way to see the relationship between mental health and lifestyle habits.

## Introduction

An increasing number of imprisoned persons will reach 50 years in Western societies in the upcoming years (1, 2), and they continue to remain a neglected sub-group among the prison population (3). Incarcerated older individuals often require special care for they are more ill than their younger counterparts (4, 5). The harsh carceral environment makes the incarcerated age more rapidly and hinders their uptake of good life habits. For example, some older adults in prison lack mobility leading to exclusion from sports and leisure facilities when compared to the younger individuals (5, 6). Likewise, no specific incentives exist for older incarcerated person's participation in sports. Furthermore, older persons often do not take actions toward improvement despite their harmful lifestyle habits in prisons (7). In medicine, lifestyle habits comprise physical activities, nutrition, and consumption of drugs, alcohol, and tobacco.

Individual and systemic factors play a role in these unhealthy dynamics. Indeed, there is no specific program for lifestyle changes in prison for older individuals, even if they suffer from several health conditions such as hypertension, arthritis, chronic back pain, COPD, cardiovascular diseases, diabetes, asthma, and liver diseases (8, 9). Depending on reporting methods, 11–46% of incarcerated individuals also have at least one mental health diagnosis (4, 8, 10, 11). However, these somatic and mental health problems could be ameliorated if specific lifestyle modification programs were implemented.

In Western detention centers, imprisoned persons often gain weight due to unhealthy diet and lack of physical activity. Countries with longer duration and increased frequency of incarceration experience a rise in health problems related to unhealthy lifestyle. This is particularly present in countries with aging incarcerated individuals, and this is recognized as an important public health issue. Arnold (12) predicted an increased incidence and related consequences of non-communicable disease with the rise of aging detained individuals.

Also, a systematic analysis of body mass index and diet revealed that even young female incarcerated persons were more likely to be obese (13). These study participants had a sodium intake two to three times higher than recommended norms (13). Other authors already wrote articles on the topic in relation to physical health (14, 15).

## Materials and Methods

### Study Design

There is a dearth of literature on mental health in prison, and even less pertaining to older incarcerated adults (16, 17). Our research is therefore explorative, since the topic has not been examined sufficiently yet, and they derive from a larger project, which aimed at understanding the older incarcerated adult's general experience on aging and mental health care in prison. This article addresses more precisely these participants' view on unhealthy lifestyle caused by imprisonment, its effect on mental health, and suggestions to make lifestyle habits better behind the bar. We follow the “Journal article reporting guidelines (JARS)” for qualitative research (18), which considered multiple qualitative guidelines when it was developed, including COREQ (19). We obtained ethics approval from our leading regional commission named Northwestern and Central Swiss Ethics Committee (translated from its German name: Ethikkommission Nordwest-und Zentralschweiz) as well as the Ethics Committee of Bern (in German: Kantonale Ethikkkommission Bern), the Cantonal Ethics Committee of Vaud on Human Research (in French: Commission cantonale d'éthique de la recherche sur l'être humain CER-VD), and Zurich Ethics Committee (in German: Ethikkommission Zürich).

### Data Collection and Identification Procedures

From Switzerland, a total of 15 prisons and forensic-psychiatric units were included in the study. The inclusion criteria are abridged in Table 1.

**Table 1:** Criteria for our study participants.

**Inclusion criteria:**	**Exclusion criteria:**
Incarcerated persons had had at least one contact with mental health services	Mental health was too instable to participate in a study
Incarcerated persons must be 50 years and older [decided on the notion of faster biological aging in prison (2, 4)]	Participation not allowed by prison responsible (e.g., due to dangerousness or solitary confinement)

A prison contact person supported the research team with the recruitment. Our sampling methodology was purposive, that is, the contact person was informed about our study criteria and he or she distributed our study information accordingly and proceeded with providing the informed consent documents to the potential participants. This person also scheduled the interviews once the prospective participant indicated his or her willingness to talk with the researchers. Once again, on the day of the interview, the interviewer informed the participant again about the objectives of the study, reassured and stated confidentiality. Refusal to participate was still possible at this point and all time during the interview process. Thereafter, written informed consent was obtained. There was no compensation for this study participation. Interviews were held in French, Germain, Swiss-Germain, or English according to the participant preference.

In total, 57 older incarcerated individuals were interviewed for our main project. One interview was carried out for each interviewee. Interviews were held between December 2017 and December 2018. The data saturation principle led our final decisions on the total number of participants (20). Meaning that no new information was attained with the latest data (20). To follow saturation, we did data analysis while collecting our data, allowing us to decide when this point was reached. Seven interviews were excluded because of their poor quality. Hence, data from 50 older incarcerated individuals are included in this article. Table 2 recapitulates participant characteristics.

**Table 2:** Study participant characteristics.

Number of interviews	• 50 interviews ° 14 forensic institutions ° 36 prisons
Participant characteristics	• 42 males, 8 females • Age range: 50–76 years (*M* = 61, *SD* = 7.34) • Sentencing: ° 41 serving measures ° 9 serving penalties
Language regions	• 31 from German-speaking • 19 from French-speaking

Interviews were done in person, in a separate room within the facility walls. We were able to assure privacy and allow the participants to speak freely during the process. Interviewers and interviewees had not met before this project. Two research assistants conducted the interviews. They were both trained in qualitative interview techniques. They were working as PhD students at the time of interviews. The discussions took 16–120 min (M = 69, SD = 26.19). Table 3 summarizes the semi-structured interview guide.

**Table 3:** Interview guide content.

**Incarcerated older persons**
• Typical activities and personal circumstances in prison and general conditions in prison circumstances • Mental health care received, experiences with access to mental health care, level of care received, satisfaction with treatment, perceived stigma due to MH issues • Perceived dual loyalty issues of the treating therapist • Aging concerns in prisons: aging in prison including serving security measures and their regular experience with risk assessment, relationships with younger incarcerated individuals, opinions on work and free time activities offered, what are their future plans

Our team transcribed verbatim all interviews in the language used by participants. Swiss German interviews were written in Standard German, which is the norm for Swiss studies. Quality and accuracy of the transcriptions were then assessed by another of our team members. Anonymization was carried out during the process of transcription. Participants did not receive the interviews for verification upon completion.

### Data Analysis

We used a thematic analysis approach during the entire process (21) following six phases: (1) acknowledging the data, (2) creating original codes, (3) finding themes, (4) reassessing the preceding themes, (5) designating and describing themes, and (6) producing results. First, eight interview transcripts were read and used for coding. Second, memos and codes were named by team members resulting in a code tree. Third, three of our team members coded the rest of the transcripts by themselves. The same people discussed important topics and themes for subsequent papers following steps (3–5). No disagreements emerged from these discussions in relation to this paper. Hence, there has been no need for an external expert to resolve disagreement. Moreover, they examined the overall findings from the project using results of all participants and comparing them for step (5). The first author of this paper completed the analysis for this article more in depth (step 5 and 6) pertaining to bad habits and mental health. All authors supported the interpretation and writing of this article and agreed to the results presented in this paper.

## Results

Both studied settings (prisons and forensic-psychiatric units) showed similar results with regard to the following topics.

### Prison as an Unhealthy Environment

#### Advanced Age and Health Issues Related to Sport in Prison

Not every incarcerated individual observes a healthy training routine. This may come from the lack of adapted sports in prison or the fear of hurting oneself. The importance of a professional guidance to encourage participation in age-appropriate physical activities transpired in our interviews. Older incarcerated individuals understood well the need for good habits. See Table 4, PQ1.

**Table 4:** Participants' quotes (PQ).

**Advanced age and health issues related to sport in prison**	
PQ1	[…] that's [sport in prison] what's missing here, and I am scared. That's why I walk and do sport, because I am scared that, at my age, that I am not able… You see that I'm worried, that my muscles will not work anymore. That's what is worrying me a bit. I don't move enough. That's it. Because my neighbor, who is also my friend, and my GP. She found me well, but she kept telling me: “I am afraid that when you'll go to prison… I am scared, because, if you don't move, you're at risk for arthrosis. There are a lot of things [diseases] that possibly you will develop.” That's why I keep on walking, I do whatever I need to keep moving. My health is good. Everything's alright. (F271)
PQ2	Then, we have to take a walk, which is from half past four until five o'clock. But there are only younger people and they all run fast. They run around for half an hour. I would prefer to take a walk outside, because it would be good for my body to move a little. To move at least a quarter of an hour every day. (D260) If it is outside, speakers would be set up—big ones. And afterwards the music hits. You can't talk with one another. You can't hear yourself […] And when it is in the gym […] I can't go to this weight room, because there's music continuously. Full volume […] you have to shout at each other in there. So, I know a lot of women now, who don't go to the weight room in here, because of this music. (D266) But they [younger incarcerated individuals] usually leave it chaotic and messy. There is filth everywhere. It is all very uncomfortable. Because if you have to clean equipment, when you want to use it and you have to clean it first; then, that's extremely uncomfortable. […] Even if, you are in a fitness center, you can't expect for weights to hang themselves where they belong. You are expected to exercise with a towel and not to let sweat dry on the benches, and so on and so on, or on the bike, and/or on the stepper. Crumbles of food and everything that crumbles is just glued and dried up with sweat…It is extremely uncomfortable! (D277)
PQ3	When I asked to order a sport mat for my back exercises and my yoga, they answered, “It has been 20 years that I work here. I have never given a dance mat. For us, it's luxury. So, NO.” “Yes, but then you tell me to do exercises,” [I answered]. “But no. I won't give it to you!”, [they said] […] I still have to FIGHT to keep doing physiotherapy. (F287) I didn't feel like going down to the room. I just didn't feel like doing strength training, which I used to do regularly at the [facility in place in German-speaking Switzerland]. There are also a few machines missing. There is no “Kieser back training” possible. The “Kieser back training” is not possible because machines are not available. That's a fact. We don't have the machines for such exercises. And I just put together an alternative—“a hotel room exercises program” last Monday. It's an alternative. It's not the best thing to do, but it's better than doing nothing. And I have to do this program regularly now. (D265)
PQ4	The pain was provoked [by a training machine]. It went away after I put some cream on. I still put on cream. “Next time,” I told myself, “I'm never going to do this training machine again.” […] Mrs. [third person], she is the one who teaches the aerobic classes, who controls a little bit [which machine we use]. She told me: “So, you don't need to respect the exact number of times [that you come to do training]. If you want to come once or twice a month, that's enough.” (E271)
PQ5	I like to do sports. But I've just told miss [name of the sports teacher] last week that I was going to start a survey. I think I would have to say (but I discussed it with her first) that maybe there are just so few opportunities for older women here. But she has explained to me, miss [name of the sports teacher], that the problem is that they never do so [have more activities for older incarcerated individuals]. Women don't come to her. They don't express what they need. I think that older women would go. Though I think it's just problematic because the music is super loud […] When you are older, you are no longer as agile as maybe a 20-year-old. You can no longer keep up, as one who is really fit. Last year, I did yoga for example. I found it good. […] But I told her [physical education teacher], I think [name of the sports teacher] should chill and use less music with a little less craziness and a bit more exercises adapted to the tempo of the older ones. …. I think that if there was an evening where only the 50+ could get into the weight room, I would think there might be more that would go. You'd have to try it out, right? (D269)
**Unhealthy prison food**	
PQ6	The food has become a catastrophe recently. When we would take the cover of the plate, it stank SO much. Even if I would have had hunger, I would never have eaten that. The meat was so old. (D270) Let's say there's fish, fried fish, spinach with potatoes: the fish would be dry like a shingle; the spinach had funny… yes, it [has] a funny taste, and the potatoes are cut in quarters. The boiled potatoes are never cooked. He will not make it so. I, just, can't. Couldn't it be done in the kitchen with a steamer? He [the cook] just couldn't cook the potatoes well (D276). The food—one has to say, “It's very, very, very (how should I say this?) shitty!”. One would say [shitty] or bad. It is simply not well-balanced. It is badly done […] Not a lot [in quantity] and all bad. It is different in other prisons, completely different. At [institution I], it would always be freshly cooked. Also, always actually fresh, 7 days a week. And there they pay attention to fresh foods, no? (D277).
PQ7	It's very vegetarian at night! And, sometimes it's undercooked. Sometimes, something's missing. It's tasteless. (F291) Well, one must say that we eat pretty badly. There are strange sauces. The meat patty is always overcooked. Happily, well, practically for every supper, I eat in my room, and my lady friend brings me home cooked meals. Yes, she brings them to me every Wednesday or Saturday. She brings stuff. (F281)
**Special diet and medical conditions—the lack of adaptation**	
PQ8	To eat, they give me also (because I have talked with my doctor, and he had communicated with the kitchen), they give me fruits and yogurt. […] Afterward, the doctor called the prison, in front of me, in his office to the kitchen, to have me a special menu. They never did it! […] There wasn't enough food in the morning, and then the doctor had to call the kitchen. Then, they start giving me a fruit sauce, and then a piece of fruit, and a yogurt. Always, always. So, I can put it in the fridge, for morning after, because I cannot eat cheese, not butter. Puff, there is many things that I cannot eat. Then, on Sunday afternoons, they give me a piece of cheese, potatoes, and all. I can't! That's why my diabetes raised during the week-ends. (F250)
PQ9	The cooking is good here [in prison]. It is. I eat everything. It is good, but one thing: there is too much fat. Cholesterol, here. Hey yes! It's VERY greasy. It's too bad! And, for me who have severe heart problems, you know, who had two heart attacks and all […] (F288) Food is bad. Then, 18 kg lost. That's bad. One year, 18 [Kg]! […] Not healthy. I can't eat. I work in custody. I'm cleaning for a day−14 francs. And then sometimes other works too. […] And then I eat too little. (D272) I am vegan. Also, I am decisive with food. Sometimes, we cook everything together. On Wednesdays, once a month, there's a cooking class. And then, it's meat that is deemed important or some other food? That doesn't go along with me naturally. This is how I feel. (But) I know that I will leave 1 day. I have a goal. Well I had well-behaved until today (D279).
**Recreational drugs in prison**	
PQ10	I have always consumed recreational drugs. I was so stoned and a lot (emphasized). At the time, I had about six, seven, or eight joints a day. Even in prison. The “walls” did nothing to stop it from coming in. Anyways, at some point, I also had a little “drug business” (small drug
	business) in prison. Somehow, I started feeling that the whole thing was getting more and more stressful. And then I actually knew I had to go to prison. I knew the problem was still small. So, talking of recreational drugs, I said, “Yes, now I'll try.” I really wanted to try a life without drugs, because I only knew life with recreational drugs. (D241)
PQ11	It definitely made it easier for me that I didn't relapse, yes. But it was actually that, I really wanted to try a life without recreational drugs now in prison. The result is just that I don't regret it. I actually live quite well, right? And because of that, I didn't see everything so narrowly nor with pink glasses […] there is recreational drug in circulation in every Swiss prison. Though, prison A, of course, was actually a paradise for drugs at the time I was there. There, you could have it all, any time, right. (D244)
PQ12	I had an acquaintance with another prison who couldn't get out of the cell. […] He had no longer a sense of purpose. He didn't want to live anymore. Then he even slipped into drugs over time. […] In prison. Yes, it doesn't take much here in prison [to let yourself go]. (D275)
**Routineness and boredom**	
PQ13	It's very much the same pattern every day but there is nothing outside of the stress you bring on yourself from being here. There is no external stress applied. […] Day after day, the repetitiveness, the lack of change. Just to keep yourself motivated enough to get up in the morning (not that you actually have the choice here because you have to work, but just to be able to do that is quite difficult). You are not given necessarily the freedom to, you know, even to indulge yourself and say, you know, “I just having a bad day, I cannot cope.” You know? “Let me just stay in bed.” It's “no sorry we do not do that here.” So, it can be very difficult. (D252)
PQ14	The main point is that, unfortunately, for almost every prison and in every cell, there is a television. Really, you watch a lot of TV [in prison]. I tried to get busy, a little bit. Yes, there is a computer that we can rent here […] Many incarcerated individuals have an old PlayStation. They play with consoles or something. They try to have fun. Sure, there are few things a bit intelligent or adequate to do to amuse an old fellow. Though that's a bit more difficult in here, really. Of course, reading, the library, logic puzzles or books and such, but now, for example, with courses or further training, such things are very, very, very difficult here, that is not necessarily encouraged and is relatively tedious (stresses) to, to, to get, to achieve or to find the support accordingly. (I: Mmh) Of course there are social workers, there are also special ones where they are responsible for how and what, but until the whole thing goes into effect and does and does is absolutely tedious anyway. (D248)
PQ15	Having possibilities also means a lot to me. It has some advantages if you get to do something with you time, or else you know. Of course, that's the thing after a while. For example, if we could do a lot of sport or just do thing by ourselves, like dealing with courses or something else. Though, that isn't the case. It's a bit of a shame. (D248)
**Lack of responsibility**	
PQ16	The biggest challenge in my situation is to put myself back to everyday life and then to know how to or to be able to manage my daily life. Then it's true that at [name of establishment X] we are a little bit taken like babies—old babies. If I find myself like now, alone in an apartment, well it will be necessary that I learn again how to conduct myself… that's a challenge! (F283) It's true that here it's a summer camp. I mean it's meant to be for kids. We would say, because they TAKE US more as kids [than adults]! This is the worst part, that they are not even able to see the difference. As I said, there's a difference between a 20-year-old kid and someone who has been independent his entire life. (F287)
PQ17	Here, the food is on the table and you have zero responsibilities, *nada*. I didn't even have to think about my appointment with you. There's a piece of paper with info on it: “Think that Mrs. [name of P], then you have to…!” It is done so. Everything is said here (emphasized). Here you will be told when to take yours tablets. Any responsibility will be taken away from you here. You can decide when you want to switch off the light, when you want to pull the curtain and when you want to switch off the television. These are the things you can decide for yourself. I also believe that it is simply not the way to succeed in life and not committing a crime afterwards. (D269)
**Regularity and fun in physical activities**	
PQ18	Yes, I do my sport. I don't do judo. I don't fight. I can't do it, but I exercise regularly because of my health. I do adequate training every day. And I keep myself so healthy. (D271) For real. It is compulsory to do sport. If not, you get sanctioned. There is inclusion. You have no wages in the afternoon. The TV is taken out. You are not allowed to do long readings. You have to go outside and if you don't. Then you have to go to the doctor for a certificate. You almost never get one of those. So, you are almost never released from sport. Though I just don't like it anymore. Everything else is so exhausting for me, I just don't like physical activities anymore. I don't like using my head anymore. (D266)
PQ19	Of course, it is clearly a bit limited here [sport with older people]. This is something that I really, really miss: just a bit of structured sport where you can do more with the older than 50. (D245) So, there is no conceptive for example art or drama or anything that may free people, that may engage them on a more emotional level. It seems to be very much brought down to sport. It seems to be the big activity, but very individual. You go to the gym, maybe you play table tennis with somebody else, but there are very few to the point of almost no group activities. As any place where you could learn something. (E252) There are team sports. We have the opportunity to do sports twice a week with the group, with the house group, with the pavilion group and that's something that is VERY, very good and should not be underestimated. The sense of camaraderie, that you can develop in such a group. The mutual cheers in the game and the joy that you can share with each other when you play with the best team. These are nice moments that we also have here. In captivity, they actually give us the opportunity to do sports twice an hour instead of working. Twice a week instead of working for an hour and a half. Of course, there is the possibility also in the evening to do recreational sports. (D265)
**Better food**	
PQ20	In the 80'… I'm here since… my first imprisonment was on [Date] 1982. At that time, we could… the food was not enjoyable. Since, then it has really, really changed for the best. More so the cooking, really. (D241) No, the food is not that bad. Nothing else is irrelevant. I am not a gourmet. But it's just fine otherwise. It is good. (D265)

There is a frequently expressed discomfort from older incarcerated individuals when it comes to doing sport with younger ones. Older imprisoned individuals reported their different needs when completing physical activity than their younger counterparts. These included their need for more time to complete a less strenuous activity, the noise levels that become unbearable for them, and the inacceptable habits of younger persons in observing general rules related to the use of training equipment. These maladaptations of prison services causes a reduction of participation in sport for elders in prison. Also, they recognize the lack thereof. See Table 4, PQ2.

Even though the lack of cleanliness and loud music affect all individuals in prison, this seems to affect older ones more specifically, according to our interviews when older interviewees talked about their younger counterparts. Asked-for equipment also seemed not to be given to incarcerated individuals. One highly motivated gentleman was able to adapt his routine to prison even when needed equipment was not available. Nevertheless, not every imprisoned individual has the knowledge and motivation to do so. Especially if there is no support and even obstacles existing in the penitentiary. See Table 4, PQ3.

As incarcerated individuals get older, they experience health problems reducing their ability to participate in physical training. One incarcerated woman expressed her problem with her knees (D266). Another imprisoned individual talked about back problems (D265). Pain may be a major factor for not doing fitness training which could be adapted in certain circumstances. See Table 4, PQ4.

Adapted sport for the elderly seems to be lacking in prison as prison is also recognized as bad for health in general. Their medical conditions should lead to a better training plan and special measures for the elderly. It seems to be known by some health professionals that prison is bad for health. See Table 4, PQ5.

#### Unhealthy Prison Food

According to most interviewees, food is not as nutritious as expected by participants and often is mediocre in taste. “The cook cannot cook!” (D276), said one of the participants. Participants also complained about the poor quality of the meals that they received. See Table 4, PQ6.

All the above were German-speaking incarcerated individuals. French-speaking imprisoned individuals also complained about the food quality and quantity. One participant said that a friend brings him food. He is lucky that he could count on the help of a friend. Though, not everyone has this chance. So, cultural aspects related to Swiss linguistic regions did not play a major role when one talked about food. See Table 4, PQ7.

#### Special Diet and Medical Conditions—The Lack of Adaptation

Health problems are not taken into consideration when adapting an incarcerated individual's diet. That means that special regimens are not followed. A medical prescription is demanded to obtain healthier food for some, but this is not always fulfilled accordingly. See Table 4, PQ8.

Participants reported that fatty ingredients are also exceedingly present in an incarcerated individual's diet even though this can potentially worsen health conditions. Moreover, incarcerated individuals stated that an unhealthy diet in prison is responsible for them developing health problems. See Table 4, PQ9.

Since good behaviors are a prerequisite for incarcerated individuals, an interviewee could not really tell authorities that he does not eat meat. As food is customary, everyone must eat meat and get along with it despite some of them being vegan or vegetarian.

#### Recreational Drugs in Prison

According to some interviewees, prison appears not to be a good time to stop recreational drugs. This partakes in the negative aspects of prison on life habits. See Table 4, PQ10.

It is uncertain if the previous succeed in stopping recreational drugs. It is unclear if it got more difficult to have recreational drugs in prison, but this incarcerated individual sees imprisonment as an opportunity to stop taking those drugs. See Table 4, PQ11.

Recreational drugs are easily available and frequently used in the penitentiary. Depending on the incarcerated individual's will to stop consuming, this may lead to worsening mental health as part of bad life habits. See Table 4, PQ12.

### Motivation Weakened by Prison

#### Routine and Boredom

Routineness and invariability are omnipresent in detention and was reported by many participants. See Table 4, PQ13.

This shows how prison routines may lead to a lack of spur even for people who used to be very motivated. Boredom is seen as continuous by some (D270 and See Table 4, PQ14).

Lack of activities is noticeable. “There is nothing for individuals, no group activities” (F285), one said. This results in boredom in prison. See Table 4, PQ15.

#### Lack of Responsibility

Also, we noted that responsibilities were taken away from incarcerated individuals. This, in turn, could worsen the lack of motivation to implement a healthy lifestyle. Imprisoned individuals are being treated like “children.” “We [incarcerated individuals] are infantilized! They [prison staff] make us IDIOT. They make us voluntarily stupid! I can't stand this!”, (F285) a participant said. They find that their autonomy is not promoted to the fullest. See Table 4, PQ16.

Incarcerated individuals have no or very little responsibility in their view. Subsequently, lack of impetus and responsibility to get through their daily routine is reflecting on incarcerated individuals' hindrance for changes in bad habits. See Table 4, PQ17.

### Guidance for Better Practice

As many incarcerated individuals complained about bad living conditions in prison pertaining to sport, food, and routineness, few expressed a different perspective. Their experience could help set the example for other facilities.

#### Regularity and Fun in Physical Activities

Regularity in physical activities integrated more easily to a daily routine renders it easy. It can be an individual choice. Although, making it a mandatory activity for all may help also to enhance sport in prison from a structural perspective. See Table 4, PQ18.

We should be careful in forcing sport on older incarcerated adults as the latter expressed his discontent. Hence, adaptations could be made for more strenuous physical activities. Walking appeared to be very popular as many imprisoned individuals explained that it is their main physical activity. “Walks maintain good health. It's well-known,” one incarcerated individual mentioned (F282). They do promenades in the courtyard which is obligatory for some (F288). As it is done more regularly, sport appears to be easily integrated in one's routine. Other positive aspects of sport could also be great motivations, apart from obligation, and routine. Incarcerated individuals suggest that sport could be an enjoyable moment between imprisoned persons, especially for older ones, if it was to be adapted to them. The spirit of communion is important in doing sport. It appeared to be an important motivation for most. Team spirit and camaraderie are reported as being very important. See Table 4, PQ19.

#### Better Food

While many incarcerated individuals voiced their aversion to prison food, others mentioned how well they eat behind bars. See Table 4, PQ20.

So very low expectations or previous experiences in prison seem to help incarcerated individuals appreciate food when in detention centers. Nevertheless, the quality and quantity have been considered very low for most of the interviewees.

## Discussion

### Summary of the Results

**Figure 1:**
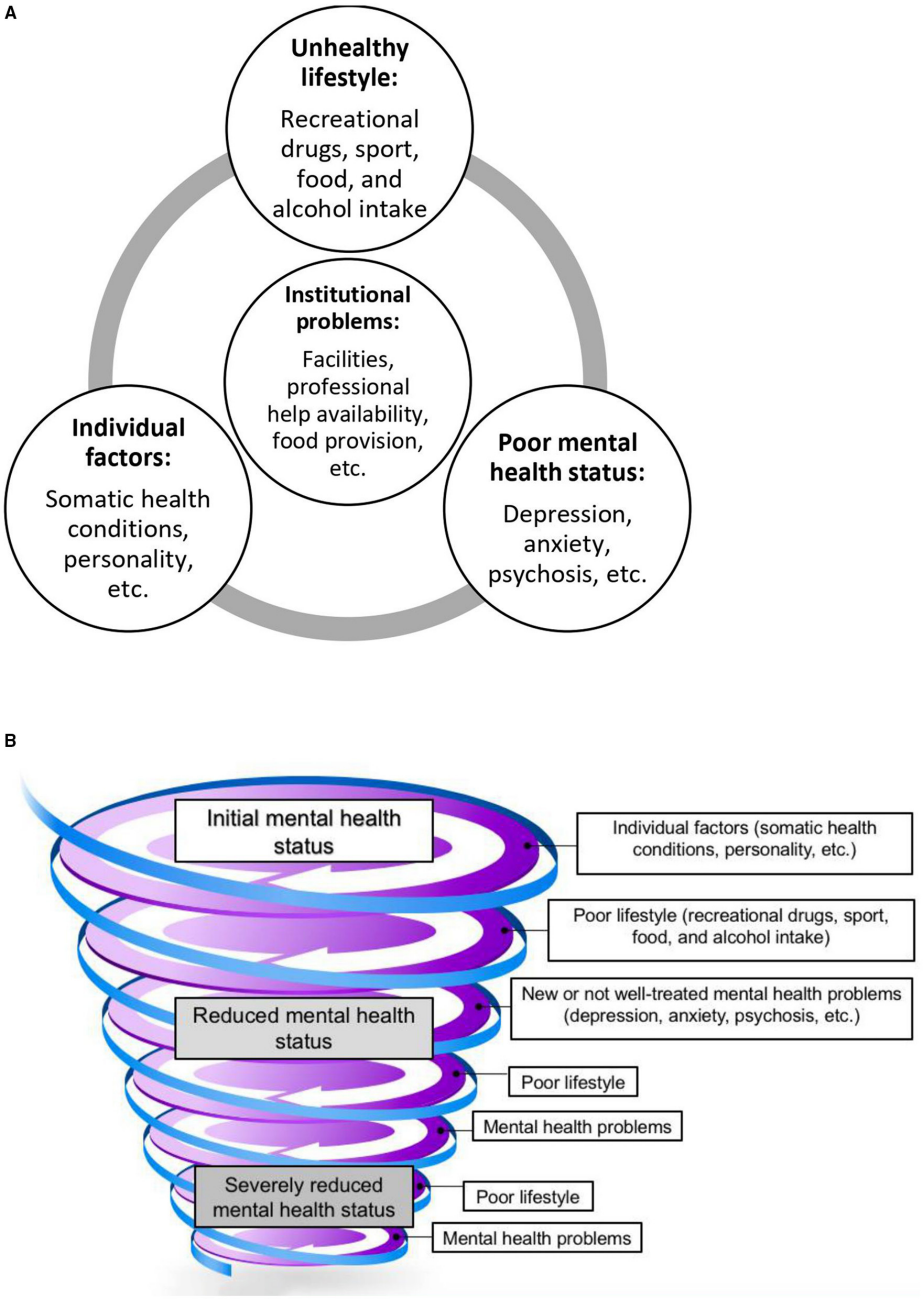
Downward spiraling of unhealthy lifestyle and poor mental health. **(A)** Interactions between factors pertaining to mental health. **(B)** Downward spiraling.

We delineate six major aspects of unhealthy lifestyle in prison. First, unadapt sport for elders prevail behind the bars. This has already been demonstrated in several articles (5, 14, 22, 23). Second, exercise equipment is either lacking or in bad condition due to poor maintenance. These two previous aspects might lead to more inactive lifestyle on the part of older incarcerated individuals, leading to health decline (5, 14). As already mentioned, thus far, neither the prison infrastructures nor the organization of activities has been designed for elderly people, neither in Switzerland nor in other countries. Third, many incarcerated individuals complained about the food quality and quantity. Food is starchy or fatty in prison according to interviewees. Furthermore, the adjustment of diets for specific medical condition is lacking (5, 16). Incarcerated individuals experience worsening of their health condition because of the preceding and might be related to earlier aging (2, 4). Forth, it is unclear if prison exacerbates drug consumption according to our participants. However, a Swiss study reported that older incarcerated individuals usually consume less drugs but more alcohol according to literature on the topic (16). Fifth, boredom and routineness do not help incarcerated individuals to make changes in their unhealthy lifestyles. Last, our results show that lack of responsibilities also hinder change for imprisoned persons. Motivation is essential to promote a change toward a better lifestyle. The monotony and lack of challenges may affect one's perspective on life changes. This may cause an unwillingness to adhere to a better lifestyle. Therefore, the penitentiary is a burden for change and contributes to a downward spiraling of bad habits. Since an unhealthy lifestyle leads to poorer mental health (15, 24) and in turn poor mental health to an unhealthy lifestyle (25), this creates a downward spiraling of unhealthy lifestyle and poor mental health (Figure 1).

### The Downward Spiraling

We need to address this pressing issue of bad habits and mental health of older adults in prison. We use a downward spiraling framework to organize our ideas as it presents a useful approach to connect different factors with one another. This framework was conceived by our team following knowledge of the recent literature on these topics, independently from our data, as a skeleton with which our data was conceptually organized. Similar to our proposed framework, another interesting model is that of Fredrickson and colleagues, who reported that the upward spirals of positive emotions counter downward spirals of negativity (26). Although it is not prison specific, it shows how psychosocial components interfere with positive and negative emotions. It also shows how a downward spiraling model can theorize psychological and psychiatric problems. Nevertheless, it lacks a few components of the spiraling model that we put forth, such as lifestyle habits and institutional factors.

Our framework (Figures 1A,B) therefore presents a useful approach to illustrate the connectedness of different factors which could consequently be investigated by future research. The pathological relationship between mental health problems and poor lifestyle is well-known (27). Sleep disorders, loss or gain in appetite, fatigue, and lack of motivation can be symptoms of mental illnesses. These can lead to a worsening lifestyle because of the lack of motivation to eat well, do sport, and be sober. The lack of adapted physical and mental activity, as well as a healthy diet in prison worsen these further. In turn, bad habits negatively impact mental health (24, 28). Worsening of mental health then leads to a decline in lifestyle habits, thus creating a downward spiraling (Figures 1A,B). Our proposed descriptive framework may allow policy makers and prison personnel to seek to address these varying concerns as a whole and not target the pieces. Taking a piecemeal approach does not produce much needed improvement in health and well-being of the prisoners, and thereby cost-efficient prison administration.

In prison, as shown by our interviewees, the environment is deleterious. It contributes to downward spiraling and hinders a lifestyle change. Poor mental health should be treated with psychotherapy in an integrative approach (29) to gain access to a healthy lifestyle modification if needed. Prison offers a unique opportunity for change. It can be health-arming or health-promoting (30). Nowadays, many mental health professionals are promoting an integrative approach to treat psychiatric conditions (27, 29, 31). These experts campaigned for the prevention and treatment of mental health conditions through healthy lifestyle such as regular physical activity, wholesome food, and sobriety. Hence, individual and institutional aspects need to be addressed to better treat mental health conditions. Indeed, the WHO report on mental illness mentioned that the mental health determinants are not only individual factors, but also environmental and socials factors (32). Consequently, poor mental health is at the interface of many determinants such as physical health, physical inactivity (24, 33), discrimination, disrespect of human rights, and many others. These factors are particularly significant and manifest in prison because the environmental factors are predominantly influential.

Even though prisons can be deleterious for the elders incarcerated there, the penitentiary has shown to have some positive aspects. In some prisons, food and exercise are well-balanced according to a few of our interviewees. One participant (D271) has shown resilience and maintained regular physical activity despite being detained, although he was the only interviewee showing such an attitude toward sport. Those examples show that change is possible, since good conditions existed in some prisons. Thus, we need to address the problem of detention institutions which are not health-promoting. Our findings are important because they present the aging imprisoned individual's perspective. Although our results are similar to what is known with similar studies on younger imprisoned individuals about psychotropic medication (34), racial disparities, and prison experience of care (35, 36), it fills in part of the well-known (by clinicians and researchers) lack of literature on the older incarcerated persons (16, 17). In doing so, we further substantiate the poor conditions of the prison infrastructure and add to the calls to improve the care given to prisoners in order to halt the downward spiraling of bad habits in prisons. Doing so will not only be beneficial for the health and well-being of the individual prisoner but also the prison system as a whole.

### Limitations

Although we sought to reduce our result biases diligently through various methods, they might still be inherent to our study and study design. First, social acceptability is a common limitation to qualitative studies. That is, participants might not say what they think but rather what they think investigators want to hear or what is socially right. To account for this bias, we assured anonymity and confidentiality to limit its impact. Second, volunteering bias might limit the strength of our results. We accounted for this through saturation. We were able to observe variation and multiple themes. Third, interviews were done in the participant language of choice and translated to English for this article. Some elements might have been lost due to translation. Our international team who encompasses French, German, Swiss-German, and English speakers reviewed translations to reduce biases due to translation. Fourth, our study included only older persons deprived of liberty with mental health problems. However, we could assume that similar factors affect everyone who is incarcerated. As our study sample is unique, it could be argued that they have more problems. If this is the case, it is important to help affected individuals even though they might be a minority. Finally, the sample is not representative of all older prisoners in Switzerland because generalization is not a goal of a qualitative methodology. Instead, purposive sampling in qualitative studies aims at collecting a high variability of opinions and different situations.

## Conclusion

Many studies on incarcerated adults and psychiatric patients have come to the same conclusion (34–36). However, there is a scarcity of research when it comes to the older imprisoned adult with mental health problems (16, 17), which our study findings address. Thus, our project gave voice to older imprisoned adults, and we have incorporated our results within this topic of bad habits in a downward spiraling framework. Prisons hinder change and worsen poor lifestyle; thus, contributing to the downward spiraling of mental health and bad habits. Because a healthy lifestyle is key for mental health care, we hope for a change of mentalities in prison. Our research outlined the problems related to lifestyle in prison. Food, sport equipment, and opportunity for change are desperately lacking behind the bars according to our participants. This would help imprisoned individuals to break out of the downward spiraling of bad mental health and unhealthy lifestyle. To paraphrase Victor's writing: the force of a democracy can be measured by the way it treats his child, his older and his incarcerated individuals (37). We hope for a change pertaining to lifestyle and mental health in prisons for the older incarcerated persons, as more and more mental health professionals are promoting a more integrative approach for the treatment of mental illnesses (27, 29, 31).

## Data Availability Statement

The raw data supporting the conclusions of this article will be made available by the authors, without undue reservation.

## Ethics Statement

The studies involving human participants were reviewed and approved by Northwestern and Central Swiss Ethics Committee (translated from its German name: Ethikkommission Nordwest-und Zentralschweiz) as well as the Ethics Committee of Bern (in German: Kantonale Ethikkkommission Bern), the Cantonal Ethics Committee of Vaud on Human Research (in French: Commission cantonale d'éthique de la recherche sur l'être humain CER-VD), and Zurich Ethics Committee (in German: Ethikkommission Zürich). The patients/participants provided their written informed consent to participate in this study.

## Author Contributions

BE and TW designed the project. HS contributed to data collection. HS, TW, and FP analyzed data of the project. Specific analysis was carried out by FP and validated by all co-authors. FP wrote the manuscript. All co-authors read draft versions of it, provided critical and useful suggestions to improve the quality and precision of our data analysis, and thus the quality of the overall manuscript. All authors approved the final version submitted for publication and take responsibility for its content.

## Funding

This work was part of the larger research project Agequake in prisons—second part: Mental health care and forensic evaluation of aging prisoners and persons serving security measures in Switzerland and was supported by the Swiss National Science Foundation (Grant Number: 166043).

## Conflict of Interest

The authors declare that the research was conducted in the absence of any commercial or financial relationships that could be construed as a potential conflict of interest.

## Publisher's Note

All claims expressed in this article are solely those of the authors and do not necessarily represent those of their affiliated organizations, or those of the publisher, the editors and the reviewers. Any product that may be evaluated in this article, or claim that may be made by its manufacturer, is not guaranteed or endorsed by the publisher.
